# Legume-rhizobia signal exchange: promiscuity and environmental effects

**DOI:** 10.3389/fmicb.2015.00945

**Published:** 2015-09-08

**Authors:** Mario A. Lira, Luciana R. S. Nascimento, Giselle G. M. Fracetto

**Affiliations:** ^1^Agronomy Department, Federal Rural University of PernambucoRecife, Brazil; ^2^National Council for Research and Scientific and Technological DevelopmentBrasília, Brazil

**Keywords:** tropical legumes, broad spectrum, soil acidity, soil temperature, salinity

## Abstract

Although signal exchange between legumes and their rhizobia is among the best-known examples of this biological process, most of the more characterized data comes from just a few legume species and environmental stresses. Although a relative wealth of information is available for some model legumes and some of the major pulses such as soybean, little is known about tropical legumes. This relative disparity in current knowledge is also apparent in the research on the effects of environmental stress on signal exchange; cool-climate stresses, such as low-soil temperature, comprise a relatively large body of research, whereas high-temperature stresses and drought are not nearly as well understood. Both tropical legumes and their environmental stress-induced effects are increasingly important due to global population growth (the demand for protein), climate change (increasing temperatures and more extreme climate behavior), and urbanization (and thus heavy metals). This knowledge gap for both legumes and their environmental stresses is compounded because whereas most temperate legume-rhizobia symbioses are relatively specific and cultivated under relatively stable environments, the converse is true for tropical legumes, which tend to be promiscuous, and grow in highly variable conditions. This review will clarify some of this missing information and highlight fields in which further research would benefit our current knowledge.

## Legume-Rhizobia Signal Exchange Importance and General Information

Biological nitrogen fixation is one of the main biological cycles worldwide (Canfield et al., 2010) and is estimated to contribute close to half (Herder et al., 2010) of the world’s biologically available nitrogen. Most of that fixed nitrogen comes from the legume-rhizobia symbiosis, which is based on a very large and constantly changing group of bacteria generically called rhizobia, including *Allorhizobium, Aminobacter, Azorhizobium, Bradyrhizobium, Devosia, Ensifer (Sinorhizobium), Mesorhizobium, Methylobacterium, Microvirga, Ochrobactrum, Phyllobacterium, Rhizobium*, and *Shinella* among the α-Proteobacteria; *Burkholderia, Cupriavidus*, and *Herbaspirillum* among the β-Proteobacteria (Vinuesa, 2015); and at least one *Pseudomonas* sp. from the γ-Proteobacteria (Shiraishi et al., 2010). This usage of rhizobia as a catch-all name has been challenged recently because it was based initially on the *Rhizobium* genus (then the Rhizobiaceae family), whereas we now know that at least three classes of the Proteobacteria include at least one genus with this capability. In contrast, this well-recognized term has been used extensively and, as such, is used throughout this review.

This symbiosis begins with an elaborate signal exchange process that is among the best studied between bacteria and plants (Hirsch and Fujishige, 2012). Initially, the legume root releases exudate compounds such as sugars, amino acids, several classes of proteins classes (De-la-Peña et al., 2008, 2010; Badri and Vivanco, 2009; Badri et al., 2009), and flavonoids, and phenolic compounds (Broughton et al., 2003), such as flavone, flavonones, isoflavones, and betains (Cooper, 2007). These compounds induce chemoostatic reactions from the bacteria and act as nodulation gene inducers (Hirsch and Fujishige, 2012; Ryu et al., 2012).

These compounds may act as weak or strong inducers, whereas others are inhibitors or have no effect on nodulation (Mulligan and Long, 1985; Firmin et al., 1986; Peters et al., 1986; Redmond et al., 1986; Hartwig et al., 1989, 1990; Hungria et al., 1992; Bolaños-Vásquez and Werner, 1997; Begum et al., 2001; Mabood et al., 2006; Subramanian et al., 2007).

Which compounds, or class of compounds, induce nodulation the strongest varies among symbiotic pairs. For common beans (*Phaseolus vulgaris*), the strongest inducers are genistein-3-*O*-glucoside, eriodictyol, naringenin, daidzein, genistein, and coumesterol (Hungria et al., 1991a; Dakora et al., 1993b); this plant also releases other classes of compounds such as anthocyanidins, flavonols, isoflavonoids, and flavones (Hungria et al., 1992). For soybeans (*Glycine max*), the most effective plant-to-bacteria signal has been variously found to be an isoflavone (Subramanian et al., 2006), jasmonic acid and its derivatives (Mabood and Smith, 2005), or genistein (Zhang and Smith, 1995).

After the nodulation genes are activated, the rhizobia release nod factors, lipochitooligosaccharides specific to each symbiotic association that are sufficient to activate nodule organogenesis at least under some conditions, and these factors may induce cellular modifications associated with early rhizobial root infection (Oldroyd and Downie, 2004; Cooper, 2007; Jones et al., 2007). In addition to the nod factors, several other bacterial compounds affect several stages of the interaction, including exopolysaccharides (EPS), lipopolysaccharides, K-antigen polysaccharides, cyclic β-glucan, high-molecular-weight neutral polysaccharides (glucomannan), and gel-forming polysaccharides (Fraysse et al., 2003; Laus et al., 2006; Downie, 2010; Janczarek, 2011).

## Signal Exchange Diversity and Legume Promiscuity

The complex signal exchange between plant and bacterial partners in symbiosis is also a key component of symbiotic specificity, which varies from highly specific to highly promiscuous. For example, although *Sinorhizobium* sp. NGR234 nodulates 232 legume species from 112 distantly related genera, with varying efficacy, some strains of *Rhizobium leguminosarum* bv. *viciae* do not nodulate pea (*Pisum sativum*) cultivars from different origins (Ovtsyna et al., 1998; Masson-Boivin et al., 2009).

The lack of effective signal exchange between legumes and bacteria precludes symbiosis establishment for incompatible partners, but in some situations, nodules may be formed in which the rhizobia do not enter, are not liberated from the infection thread, or do not fix nitrogen (Miller et al., 2007). This lack of recognition may occur even after the initial signal exchange. For example, *R. leguminosarum* bv. *trifolii* (*Rlt*) strain ICC105 does not fix nitrogen with white clover (*Trifolium repens*), whereas this strain is effective when paired with Caucasian clover (*T. ambiguum*). According to Miller et al. (2007), this difference is due to a region between the *nifH* gene and the *fixA* promoter that is differentially activated when in symbiosis with the two *Trifolium* species. It is not clear if this difference is due to positive or negative regulation by a specific plant signal, nor is it clear how NifA activity is regulated (Miller et al., 2007).

The combination of a vast range of compounds secreted by both plants and bacteria is one of the main characteristics of this symbiotic compatibility. Because the first step is exudation by the plant, this step may be considered the most important one. These exudates are continuously secreted into the rhizosphere, but both the number and concentration of these compounds increases when compatible bacteria are detected by the plant (Zaat et al., 1989; Dakora et al., 1993a,b; Hassan and Mathesius, 2012).

These plant-bacteria signals activate three main groups of nodulation genes in the bacteria: the common *nodABC* genes that are present in almost all rhizobia (the exception being some photosynthetic bradyrhizobia and some *Burkholderia*, Giraud et al., 2007) and are required to produce the basic structure of the nod factors; host-specific *nod* genes that are linked to specific modifications of the basic nod factor structure that allows for symbiotic specificity, such as *nodEF, nodG, nodH, nodPQ*, and *nodRL*; and regulatory genes that are linked to the activation and transcription of both the common and specific *nod* genes (Horvath et al., 1986; Göttfert et al., 1990; Lerouge et al., 1990; Sanjuan et al., 1994; Moulin et al., 2001; Schlaman et al., 2006).

Nod factor perception is mediated by Nod factor receptors (NFRs), which are serine/threonine kinases that are located in the plasma membrane and that contain LysM motifs in their extracellular domains (Limpens et al., 2003; Madsen et al., 2003; Radutoiu et al., 2003; Arrighi et al., 2006). These NFRs correspond to the Nod factor structure and act as host determinants for symbiotic specificity. This specificity was shown by the transfer of *Lj-NFR1* and *Lj-NFR5* to *Medicago truncatula*, which enabled nodulation by the *Lotus japonicus* symbiont *Mesorhizobium loti* (Radutoiu et al., 2007); the specificity of two *Lotus* species is the function of a single amino acid residue in one of the LysM domains of Lj-NFR5 (Radutoiu et al., 2007).

In addition to Nod factors, some rhizobia secrete proteins that are involved in nodulation via a type III secretion system (T3SS; Fauvart and Michiels, 2008; Deakin and Broughton, 2009). These proteins, called nodulation outer proteins (Nops), are believed to contribute to legume immune response suppression or to modulate root cell cytoskeletal rearrangement during nodule development (Bartsev et al., 2004; Skorpil et al., 2005; Soto et al., 2009). The *nopP* and *nopL* genes are found in *Rhizobium* sp. NGR234, *Sinorhizobium fredii* and *Bradyrhizobium japonicum* and are absent in pathogenic bacteria (Deakin and Broughton, 2009). In *Rhizobium* sp. NGR234, these genes are required for the nodulation of the tropical legumes *Tephrosia vogelii* and *Flemingia congesta* (Marie et al., 2003; Skorpil et al., 2005). Moreover, the nodulation of *Vigna unguiculata* by *S. fredii* is also affected by Nop proteins injected by *S. fredii* in a T3SS-dependent fashion (Schechter et al., 2010), but further studies on their effects on host specificity are still necessary.

Exopolysaccharides, bacterial cellular wall constituents, are also known to have important effects on symbiosis. For example, a defect on the EPS surface may induce failures both in the early and late stages of symbiosis, such as those observed in strains of *S. meliloti* presenting normal nodules in some ecotypes of *M. truncatula* but defective nodules in others, and this pattern may be transferred by a change in the EPS biosynthesis locus (Simsek et al., 2007). Because *M. loti* EPS mutants result in nonfunctional nodules in *L. leucocephala* but functional ones in *L. pedunculatus*, the EPS surface has also been linked to specificity in the nitrogen fixing phase (Hotter and Scott, 1991), as demonstrated by a *B. japonicum exoB* mutant fixing nitrogen in *G. max* but not in *G. soja* (Parniske et al., 1994) or some *R. leguminosarum* LPS mutants fixing nitrogen in peas (*Pisum sativum*), whereas other mutants do not (Kannenberg et al., 1992).

One point that deserves attention is the almost complete lack of literature on this signal exchange in tropical legumes, which are typically more promiscuous than temperate ones. Because of this knowledge gap, it is not known how the degree of promiscuity of a legume affects the signal exchange process because with the exception of *Phaseolus*, the best-studied legumes are all generally considered to nodulate with a few species or genera at the most (Michiels et al., 1998; Martínez-Romero, 2003; Rodríguez-Navarro et al., 2011; Rufini et al., 2013). A synthesis of a large portion of the literature identifying seed or root exudate compounds with known nod-gene activating properties (**Table 1**) indicates that more promiscuous (or less-selective) legumes may exhibit a broader range of these compounds, as per a comparison between *P. vulgaris* and *G. max*, which are less and more selective, respectively, for the rhizobial partner of the symbiosis. In contrast, the only paper we could find on *V. unguiculata* identifies only three compounds, although it has a very broad range of rhizobial partners. One further puzzle is that genistein is a known inducer for *G. max, P. vulgaris*, and *V. unguiculata*, although the rhizobia of these three species are not identical.

**Table 1:** Seed and root exudate compounds with known *nod* gene-activating factors, from legumes with broad or narrow ranges of symbiotic compatibility.

Species	Source	*nod* gene- activating factors	Source
*Glycine max*	Root exudates	Daidzein, genistein, coumestrol, isoliquiritigenin	Kape et al. (1992), Pueppke et al. (1998)
*G. max*	Seeds	Daidzein, genistein	Pueppke et al. (1998)
*Medicago sativa*	Seeds	Chryoseriol, luteolin, liquiritigenin	Maxwell et al. (1989), Hartwig et al. (1990)
*M. sativa*	Root exudates	4,7-dihydroxyflavone formononetinin	Maxwell et al. (1989)
*Phaseolus vulgaris*	Root exudates	Genistein, eriodictyol, naringenin, daidzein, coumesterol	Davis and Johnston (1990), Hungria et al. (1991b), Dakora et al. (1993b)
*P. vulgaris*	Seeds	Unidentified isoflavone, delphinidin, petunidin, malvidin, myricetin, quercetin, kaempferol	Hungria et al., 1991a
*Vigna unguiculata*	Root exudates	Daidzein, genistein, and glycitein	Dakora (2000)

A lack of depth in the literature on this topic leads to ambiguity in how to relate legume promiscuity (or specificiticity) with the signal exchange process, although this relationship is expected to exist due to the specific nature of this exchange. Thus, this relationship might be an interesting line of future research; a better understanding of this relationship may lead to biotechnological approaches to enhance or reduce the compatibility profile of a given legume similarly, to soybean breeding for broad bacterial compatibility in Africa (Gwata et al., 2005).

## Environmental Effects on Signal Exchange

Although the interaction between environmental stresses and legume-rhizobia signal exchange has been investigated, as will be discussed, these studies have also centered on temperate climate pulses, and their stresses. Much work is still needed to understand how the signal exchange process of other legumes is affected by their more typical stresses.

### Temperature

Much research has examined low root zone temperatures and their effects on signal exchange and nodulation, particularly in soybeans, but little is known about the effects of high root zone temperatures.

Low root zone temperatures inhibit the synthesis and secretion of plant-to-bacteria signals, as shown in *G. max*, in which the root exudation of genistein is strongly reduced below 17.5°C (Zhang and Smith, 1994, 1996a; Zhang et al., 1995; Pan and Smith, 1998). Low root zone temperatures also reduce nod factor synthesis and/or excretion in *R. leguminosarum* bv. *trifolii* (McKay and Djordjevic, 1993) and *B. japonicum* (Zhang et al., 2002). The molecular basis of this effect indicates that the *T3SS* gene cluster was progressively activated as temperatures increased, whereas the *nod* genes were rapidly induced at 15°C (Wei et al., 2010). Genistein has been proposed to induce this gene cluster through a regulatory cascade involving NodD1 and NodW (Krause et al., 2002).

These signal exchange effects combine to delay nodulation onset (Pan and Smith, 1998) and reduce the nodule growth rate, leading to smaller nodules (Lira Junior et al., 2005). Further confirmation that these stresses are directly linked to signal exchange is that the exogenous application of genistein is sufficient to mitigate a delay in nodulation under environmental conditions in which the root system temperature is below this threshold and the shoot is above it (Zhang and Smith, 1995, 1997; Pan et al., 1997). This mitigation is stronger for lower soil temperatures or stronger stresses (Zhang and Smith, 1996b).

### Salinity

Although salinity is known to affect Nod factor production by *R. tropici* CIAT 899 in the presence of apigenin (Estévez et al., 2009), there are indications that high salt concentrations may induce *nod* genes even in the absence of flavonoid inducers (Guasch-Vidal et al., 2012).

However, increased salinity reduces Nod factor production by *S. arboris*, which nodulates *Acacia* and *Prosopis*, both of which are legume trees tolerant to salt stresses (Penttinen et al., 2013). Similar effects were found for *R. tropici* and *R. etli*, which nodulate *P. vulgaris* (Dardanelli et al., 2012).

Similarly, to what is observed at low soil temperatures, as previously described, some of the salinity effects may be reduced if the bacteria are pre-incubated with their respective legume signals, such as genistein for *B. japonicum* (Miransari and Smith, 2009) or hesperetin and apigenin for *R. tibeticum* (Abd-Alla et al., 2013).

### Soil pH

Soil pH affects symbiosis in several ways, including signal exchange (Hungria and Vargas, 2000). For example, both *G. max* and *P. vulgaris* isoflavonoid exudation from roots were reduced when the pH was lowered from 5.8 to 4.5 (Hungria and Stacey, 1997), and some nodulation genes, including *nodA*, are inactivated by reducing the pH in *R. leguminosarum* bv. *trifolii* (Richardson et al., 1988a,b). The production and excretion of Nod factors were also reduced in acidic soils (McKay and Djordjevic, 1993).

Another effect is a change in the profile of the Nod factors secreted by *R. tropici* CIAT 899, which is tolerant to acid conditions. A total of 52 different molecules were produced under an acidic pH and 29 at a neutral pH; only 15 are common to both conditions (Moron et al., 2005). This phenomenon might be linked to the reduction in *nodC* expression by the *Arachis hypogaea* bacterial symbionts under acidic conditions (Angelini et al., 2003).

In contrast to what is observed for low soil temperatures and salinity, the addition of flavonoids did not reduce the effects of low pH on acid-sensitive or acid-tolerant *A. hypogaea* (Angelini et al., 2003), which was apparently due to increased flavonoid uptake and toxicity.

Low pH also activates a systemic, shoot-controlled, and GmNARK-dependent (Nodulation Autoregulation Receptor Kinase) mechanism that negatively regulates initial nodule development in soybeans (Lin et al., 2012), as confirmed by the reduced expression of the *GmENOD40b*, *GmNIN-2b*, *GmRIC1*, *GmRabA2*, and cytochrome P450 genes, which are critical to early nodulation stages.

### Iron and Phosphorus Deficiency

The legume-rhizobia symbiosis demands high levels of iron due to its inclusion in the compositions of leghemoglobin, nitrogenase, and cytochromes (Brear et al., 2013). Iron deficiency effects vary between legume species and may include altered nodule initiation, as seen in *Lupinus angustifolius* L. (Tang et al., 1990), or late development, as seen in peanuts (*A. hypogaea*), common beans (*P. vulgaris*), and soybeans (O’Hara et al., 1988; Soerensen et al., 1988; Slatni et al., 2011).

Iron absorption regulation by rhizobia in culture media has been extensively researched, and iron-responsive transcription regulators such as IrrA and RirA and the genes they control under iron deficiency and sufficiency have been determined (Viguier et al., 2005; Todd et al., 2006). Several of these genes encode siderophore production, heme biosynthesis, and transporters, such as the ferric siderophore ATP-binding cassette (ABC)-related genes.

Under iron-limiting conditions, free-living rhizobia express TonB-dependent receptors after activation by an iron regulator (Small et al., 2009), although bacteriod active siderophore transport is not necessary for symbiosis (Chang et al., 2007; Small et al., 2009). Mutations in ABC transporters, TonB-dependent receptors and TonB do not affect symbiosis establishment (Lynch et al., 2001; Nienaber et al., 2001), suggesting that bacteriods do not require a high affinity for siderophore absorption to obtain iron during symbiosis (Brear et al., 2013), although *S. meliloti* strains deficient in the siderophore absorption system exhibited lower nodule occupation rates under iron-deficient conditions than the corresponding wild types (Battistoni et al., 2002).

N_2_ fixation has a high energy cost, and P deficiency is an important restriction for legume production, particularly in the low-P soils of most tropical regions (Sulieman and Tran, 2015). Organic phosphates are the main source to sustain nodule symbiotic activities (Li et al., 2012), and several genes involved in recycling P are up-regulated under low-P conditions (Hernandez et al., 2009), particularly those encoding acid phosphatases (Maougal et al., 2014; Zhang et al., 2014).

Generally, the specific activity of acid phosphatases in nodules strongly increases when P supply is reduced in the growth medium but is stable when P supply is high (Araujo et al., 2008). The expression of several genes of the purple acid phosphatase GmPAP family was highly induced in soybean nodules under low-P availability (Li et al., 2012); the expression of phytate and phosphoenol pyruvate phosphatase was also increased in nodules under these conditions (Araujo et al., 2008; Bargaz et al., 2012). Acid phosphatases may have multiple functions, such as carbon metabolism, nodule permeability for O_2_ diffusion, and oxidative stress attenuation (Sulieman and Tran, 2015), which makes their study both more challenging and necessary.

### Drought and Flood

The current literature lacks information on the effects of either drought or flooding on legume-rhizobia signal exchange, although both situations are well known to reduce nodulation and nitrogen fixation (Arayangkoon et al., 1990; Marcar et al., 1991; Purwantari et al., 1995; Hatimi, 1999). Thus, further research is necessary on this topic. Nodule formation ceases completely under sufficiently long or severe drought conditions, and nitrogenase and nodule respiratory activities are also strongly diminished in soybeans and common beans (Gerosa-Ramos et al., 2003). In alfalfa, such nitrogenase activity reduction has been linked to diminished bacteriod metabolic capability and oxidative damage to nodule cell components (Naya et al., 2007).

At the other extreme, several legumes are highly sensitive to water-logged conditions, with nodule development and function being more impaired than infection. Some of these effects, including nitrogenase activity, may be even stronger than observed for drought conditions. This phenomenon appears to be mostly linked to reduced O_2_ availability (Andres et al., 2012).

### Heavy Metals and Pesticides

Although the literature contains little information on the effects of pesticides and heavy metals on signal exchange, some *in vitro* work with 30 different pesticides and environmental contaminants showed that *S. meliloti* NodD was affected, delaying nodulation, and reducing biological nitrogen fixation by *M. sativa* (Fox et al., 2001, 2004). *M. sativa* and *G. max* fungicide-treated seeds also exhibited reduced *nod* gene activity for their respective partners (Andrés et al., 1998).

More recently, it has been shown that *R. alamii*, an EPS producer, modulates its metabolism in response to cadmium (Schue et al., 2011) through the activation of biofilm formation, both in the wild type and in EPS-deficient mutants, which may reduce the effects of this heavy metal.

## Overall Synthesis

Although signal exchange between legumes and their bacterial symbionts is a well-studied process, much still needs to be clarified, particularly in relation to tropical legumes, which have been barely studied, and environmental effects other than low soil temperature.

Under at least some conditions, a delay in nodulation onset and, therefore, biological nitrogen fixation may be reduced by the exogenous supply of the appropriate legume signal. Because current predictions indicate a probable reduction in global agricultural season lengths, this phenomenon should receive increased attention.

Another field that deserves more attention is the study of signal exchange with non-traditional rhizobia, such as *Burkholderia* and *Cupriavidus*, and its effects on the plant host, for which no literature was found.

## Conflict of Interest Statement

The authors declare that the research was conducted in the absence of any commercial or financial relationships that could be construed as a potential conflict of interest.
